# A systematic literature review of requirements engineering education

**DOI:** 10.1007/s00766-022-00381-9

**Published:** 2022-05-19

**Authors:** Marian Daun, Alicia M. Grubb, Viktoria Stenkova, Bastian Tenbergen

**Affiliations:** 1 paluno-The Ruhr Institute for Software Technology, University of Duisburg-Essen, 45127 Essen, Germany; 2 Department of Computer Science, Smith College, Northampton, MA 01063 USA; 3 Department of Computer Science, State University of New York, Oswego, NY 13126 USA

**Keywords:** Requirements engineering, Requirements engineering education, Systematic literature review, Learning outcomes, Pedagogy

## Abstract

Requirements engineering (RE) has established itself as a core software engineering discipline. It is well acknowledged that good RE leads to higher quality software and considerably reduces the risk of failure or budget-overspending of software development projects. It is of vital importance to train future software engineers in RE and educate future requirements engineers to adequately manage requirements in various projects. To this date, there exists no central concept of what RE education shall comprise. To lay a foundation, we report on a systematic literature review of the field and provide a systematic map describing the current state of RE education. Doing so allows us to describe how the educational landscape has changed over the last decade. Results show that only a few established author collaborations exist and that RE education research is predominantly published in venues other than the top RE research venues (i.e., in venues other than the RE conference and journal). Key trends in RE instruction of the past decade include involvement of real or realistic stakeholders, teaching predominantly elicitation as an RE activity, and increasing student factors such as motivation or communication skills. Finally, we discuss open opportunities in RE education, such as training for security requirements and supply chain risk management, as well as developing a pedagogical foundation grounded in evidence of effective instructional approaches.

## Introduction

Requirements engineering (RE) is commonly accepted as the foundation of high-quality software [132]. Requirements engineering education (REE) must not only deal with teaching students how to specify formal and informal requirements but also how to elicit and negotiate requirements involving multiple sources—particularly human stakeholders. Thus, REE must make students aware of socio-technical challenges and teach human-related aspects, which poses significant challenges for REE in higher education.

Furthermore, students must be adequately prepared to take on industrial challenges [172, 195], while incorporating the theoretical concepts underlying RE [30]. REE is at best an afterthought in many university software engineering curricula [179], focusing on lecture-style instruction with few or no realistic examples. In many cases [64, 67], RE is not instructed in dedicated courses, but instructed as part of a generic software engineering course. The problem with this situation is twofold: on the one hand, graduates only gain a rudimentary understanding of the minimal RE knowledge required by accreditation standards [1], standard curricula [74, 75, 90], and bodies of knowledge [20]. On the other hand, the opportunity is lost to give students enough experience to pick the right RE tools for each development project. In consequence, it is left to the industry to adequately train their staff to be effective in RE.

We need a clear understanding of what to teach (i.e., learning objectives), as well as what educational approaches are the most effective, who the learners are, and what learning outcomes to strive for. Herein, we contribute a systematic literature review of the field of Requirements Engineering Education. Our work allows researchers to gain an overview of the current state of the art and provides educators with insights on how to teach which RE technique.

We consider three research goals:

- Goal 1: develop a systematic map of the current state of REE research;
- Goal 2: report on current practices and their learning outcomes; and
- Goal 3: evaluate how REE has changed over the previous decade.

We further elaborate on these goals in Sect. 3.

This paper is structured as follows. Section 2 discusses meta-studies on the field of REE. Section 3 details our research questions and methodology. In Sects. 4–6, we discuss results pertaining to each of our research goals (goal 1–goal 3). Section 7 concludes this paper.

## Background and related work

### Challenges in mastering requirements engineering

Requirements Engineering (RE) is a socio-technical, iterative process to elicit, document, and manage the requirements of a system under development [56]. RE bridges the gap between human users, developers, and managers, i.e., between people with and without software engineering expertise. RE helps to understand *what* problem needs to be solved by a (software) system. In addition, it helps to discover *who* needs to be involved in the engineering process (i.e., stakeholders) and *how* the problem could be solved by exploring trade-offs and alternatives [186]. RE requires analysis of both the problem space (i.e., context analysis) and solution space (i.e., the intervention). This is accomplished through a variety of *requirements discovery* or *requirements gathering* techniques, including eliciting requirements by interviewing stakeholders or by analyzing existing systems, before documenting the requirements in the form of a specification.

For example, interview techniques alone demand careful selection, as stakeholders may respond differently depending on the mode of inquiry. Imagine a focus group for a new mobile app to allow children to self-monitor health symptoms. A focus group consisting of physicians and children might quickly arrive at decisions about the app’s medical goals, but neglect the children’s perspective because in this setting, the children themselves might be too intimidated to contribute. Documentation techniques require similar careful choice. Storyboards, personas, user interface mock-ups, and natural language requirements (constrained or not), are useful to communicate ideas quickly with a broad audience of non-technical stakeholders, but lack precision for safety-related applications. Formal methods are very precise; however, they require substantial technical expertise and are generally unfit for directly communicating design choices and alternative solutions to stakeholders.

Despite excellent work in the field, elicited and documented requirements artifacts are often incomplete, conflict with one another, and/or suffer from other inadequacies [55, 120]. The quality of how the RE process is conducted immediately impacts the quality of the requirements, which in turn, impacts the quality of the system under development. The RE process must be iterative and perpetually monitored with regard to elicitation, documentation, and validation, as well as tracing [148] requirements from their “source” (e.g., stakeholders, but also laws and standards) to their “destination” (e.g., their refinement into more requirements, analysis results, or their implementation into code). These challenges motivate us to investigate the landscape of RE approaches as it relates to education and training.

Mastering Requirements Engineering is not only a monumental task for the learner, but also for the educator [39]. On one hand, the theory behind concepts, techniques, and ontologies is quite technical and demands a high amount of rote memorization [31]. On the other hand, most of the RE process is “learning by doing,” i.e., the learners must to experience it for themselves [65] before being able to appreciate (and with repeated exposure, eventually master) the RE process and develop a “feeling” when certain techniques are preferable over others. This dichotomy requires a carefully calibrated RE curriculum that balances theory instruction and process exposure.

### Studies on the state of requirements engineering education

In a recent REFSQ conference keynote,^1^ Martin Glinz provided a survey spanning the past several decades on RE Education literature. Indeed, over the past 20 years, a series of reports have been published into the state of the art of software engineering education that are more or less concerned with aspects of requirements engineering education. One of the earliest ones by Shaw [162] came at a time, where software engineering education was mostly done at the graduate level, aiming to prepare future PhD students. Shaw picked up the claim made in [183], where graduate and postgraduate software engineering education starts too late and should begin at the undergraduate level alongside traditional computer science education. To this end, Shaw identified “forces” impacting the software engineering industry and academia, and derived “aspirations” for higher education in software engineering to strive towards. Shaw took a wider view than RE education alone, and she aspires for software engineering education to include the need for novice software engineers to specialize into roles and sub-fields like requirements engineering, testing, and even safety assessment. Moreover, Shaw suggested that software engineering education takes an experience-based stance to allow the learner to put theory into practice and develop an intuition for the application of techniques.

By 2008 [105], software engineering curricula became relatively wide-spread at the undergraduate level, and with it came an increased focus on RE education. As pointed out by Regev et al. [146], undergraduate RE education was slow to address Shaw’s aspirations, due to discrepancies between typical project-based learning in higher education and actual industry experiences. According to Regev et al., academic classroom projects translate poorly to the industry because of their “sterile” nature, which inadequately reflect industrial practices. The authors attributed this discrepancy to the fact that academic projects must be narrowly scoped to be completed within one semester, by a few students who do not have prior knowledge of the application domain. Additionally, instructors must provide the same experiential opportunity regardless of student background and possible arising group conflicts. Regev et al.’s observations are consistent with views previously reported by a series of other authors, including [27, 33, 54, 68, 169], and later confirmed with an empirical study by Menon et al. [112].

Three systematic mapping studies were conducted between 2012 and 2020, which consist of the work by Malik and Zafar [105], the aforementioned work by Idri, Ouhbi, et al. [71, 135], and the work by Cico et al. [24]. While the mapping studies by Malik and Zafar as well as by Cico et al. take a wide aim on software engineering education at large, the work by Idri, Ouhbi et al, focuses particularly on RE education. Interestingly, Malik and Zafar report that while some of the mapped primary studies are concerned with project-based learning, the vast majority are concerned with educational technology and tools. Moreover, none of the 70 studies mapped by Malik and Zafar could be easily classified into the knowledge area “Requirements Engineering” according to the reference curricula available then (i.e., “Knowledge Area A” in [2] or “Knowledge Area C” in [90]). This indicates that REE research was incongruent with reference curricula and software engineering education research largely ignored RE as a topic. The more focused mapping study conducted by Ouhbi et al. [71, 135] reveals a similar trend: only 19 out of 79 mapped primary studies mention reference curricula. The vast majority of papers (77%, see [135]) present solution approaches with mostly graduate or undergraduate students, with only a minority describing some evaluation of existing approaches. Only few primary studies concerned with industrial training or industrial case studies were found (i.e., 16% and 6% or selected studies, respectively). Moreover, while Ouhbi et al. found that 16% of selected primary studies were written with industrial training consultants as co-authors, neither  [71] nor [135] report on industry-readiness of learners.

In summary, past studies investigating the state of the art of REE have been conducted and published in loose intervals. As the newest REE-specific study conducted by Ouhbi et al. [71, 135] stems from 2012, we expect the field to have evolved in light of the strong evolution of the field driven by new technologies (cf. [193]). Therefore, in this paper we want to provide an up-to-date investigation of the current state of REE. We investigate how the field has changed since the investigation of Ouhbi et al., and whether needs posed by new technologies have already been considered in REE research. In addition, we derive common practices and provide guidelines for REE synthesized from the found studies, which has not been done so far. Thus, we review educational approaches that foster learning objectives suitable to the requirements-related problem to be instructed.

## Research method

**Table 1:** Research questions

No.	Research question	Main motivation
RQ1	Who are the **most active researchers** in terms of publication output in the area of requirements engineering education?	Answering RQ1 helps us identify if certain researchers or groups of researchers who dominate the field of requirements engineering education.
RQ2	What established research **author networks** do exist in terms of co-authorship?	Answering RQ2 helps us identify core groups of researchers and research practices in requirements engineering education.
RQ3	What are the **top venues** for research on requirements engineering education?	Answering RQ3 helps us identify where research on requirements engineering education is being published.
RQ4	What is the number of published **papers per year** in the area of requirements engineering education?	Answering RQ4 helps us identify trends in the popularity of research on requirements engineering education.
RQ5	What are the **top cited publications** in the area of requirements engineering education?	Answering RQ5 helps us identify the most influential papers on requirements engineering education.
RQ6	What are the most commonly used **research methods** in the area of requirements engineering education?	Answering RQ6 helps us gauge the maturity of the field, as it reveals if suggested research methods have been implemented and evaluated in practice, etc.
RQ7	What are the most common **contributions** in the area of requirements engineering education?	In addition to the following Research Questions on Involvement of REE Research: Answering RQ7 helps us identify the focus of re-search activities in the field, as it reveals if researchers focus on the development of methods, tools, processes, etc.
RQ8	What are the most commonly used **keywords** to describe research on requirements engineering education?	Answering RQ8 helps us identify which topics are associated with requirements engineering education. Papers not indicating keywords will be excluded from analysis regarding this question.
RQ9	Who are the **learners** (i.e., high school students, freshman, sophomore, junior, senior, or graduate university students, or industry professionals)?	Answering RQ9 helps us determine the specific target of education approaches and thereby estimate the time when RE is introduced into the curriculum.
RQ10	What are the most commonly used **educational approaches** and what requirements engineering topics (i.e., **learning outcomes**) are instructed?	Answering RQ10 helps us gauge the heterogeneity of RE education practice.

In this section, we first elaborate on our research goals introduced in Sect. 1, and introduce the research questions explored in our systematic literature review (SLR). We then describe our SLR methodology in detail, including how we searched for relevant papers, extracted knowledge, and analyzed data.

### Goals and research questions

As mentioned in Sect. 1, this SLR complements the mapping study by Ouhbi et al. [135]. Ouhbi et al.’s work mainly investigated the type of contribution, without placing a clear focus on learning outcomes. We, therefore, provide an overview of existing research about the state of REE and its impact on students’ learning outcomes with the study at hand.

We define three overall goals for our study:

- Goal 1: Provide a systematic map of the current state of research in requirements engineering education. Such a systematic map helps researchers in relating their own research to the state of the art and educators in selecting existing approaches for application and adaptation to their own needs.
- Goal 2: Provide a synthesis of the current practices the studies reported in the systematic map (i.e., goal 1). This helps to identify approaches best suited for specific RE learning outcomes and challenges in teaching RE.
- Goal 3: Evaluate how the state of REE has changed over the last decade since the investigation by Ouhbi et al. [135].

To fulfill these goals, we defined ten detailed research questions, that allow us to assess the state of REE research. The research questions are listed in Table 1.

To achieve goal 1, our SLR contains a systematic map that adheres to established research questions for systematic maps as defined by Petersen et al. [142]. These research questions have been adapted to account for research on REE. As commonly done in systematic mapping studies, we are interested in the researchers involved in REE (RQ1-2), the major publications and venues in the area (RQ3-5), and how do authors conduct and describe their research in this area (RQ6-8). In addition, we defined research questions regarding the educational approaches used, learning outcomes addressed, and the RE techniques taught (RQ9-10).

To achieve goal 2, we relate answers from goal 1 with one another. This allows investigating the instructional theories underlying REE, with a focus on learning outcomes. Taking this as a starting point we synthesize the findings, contributions, benefits, and shortcomings of the papers in the so created sets.

To achieve goal 3, we defined the research questions to be investigated on the basis of the research questions used by Ouhbi et al. [135]. This allows us a fair comparison of our findings—particularly newer publications—with the findings of Ouhbi et al.. Thereby, it can be investigated whether the state of REE research has changed over the last decade.

**Table 2:** Inclusion and exclusion criteria

Inclusion criteria	
Published in a peer-reviewed journal or conference/workshop proceedings	
Focus on requirements engineering education	

Exclusion criteria	
Papers not focused on education	
Papers about requirements for engineering education	
Focus on requirements engineering for education systems	
Introductory papers for special issues, conferences, or workshops	
Publications shorter than three pages	
Publications not written in English	
Full text neither available online nor via interlending	

### Search procedure

The selected search method of an SLR may impact the found results considerably: manual search, database search, and snowball search may result in paper sets with significant disparities [21]. In order to avoid limiting the scope of investigation to selected venues (like in manual search), or getting “stuck” in local cliques of mutually referencing papers (like in snowball searches), we used a database search to cover the overall spectrum of possible approaches.

In this spirit, we also used broad search terms to lower the risk of missing relevant papers. Our defined search string is as follows:

TITLE-ABS-KEY (“Requirements Engineering” AND “Education”)

For database searches it is common to include synonyms in the search string; however, this was not appropriate in the case of our investigation. We excluded “training” and “learning” from the search string as pilot testing the search string yielded an extra-proportional number of machine learning and artificial intelligence approaches being included in the results, which are beyond the scope of this study. We restrained the string from including the different areas of RE as substitute for the term “requirements engineering.” Doing so would have led to a misrepresentation of the field as many techniques relevant for requirements engineering education are used in other fields. Instead, we wanted to represent what authors believe is requirements engineering education. Thus, we restricted the search to requirements engineering education literature.

In addition, we analyzed the search string using comparison by manual search for selected venues (as suggested as part of the quasi-gold standard, cf. [197]). Analysis showed high sensitivity of the search string.

We used Scopus for the search because it covers many publishers, including the most common publishers for computer science research (e.g., ACM, IEEE, Elsevier, Springer), and unlike Google Scholar allows filtering non-peer-reviewed publications. The search string was developed based on the literature review’s topic and research questions, as is commonly done in systematic literature reviews [89, 142].

### Study selection

The search was conducted by three different researchers who evaluated each paper based on the inclusion and exclusion criteria (see Table 2) on their own. We considered papers published at any time until December 31, 2020. Papers were *included* in the set of relevant papers of the respective literature review if all researchers found the paper relevant and *excluded* if all found the paper irrelevant. In cases of inconsistent perceptions of the paper’s relevance, the paper was discussed among the researchers until consensus was reached.

**Fig. 1:**
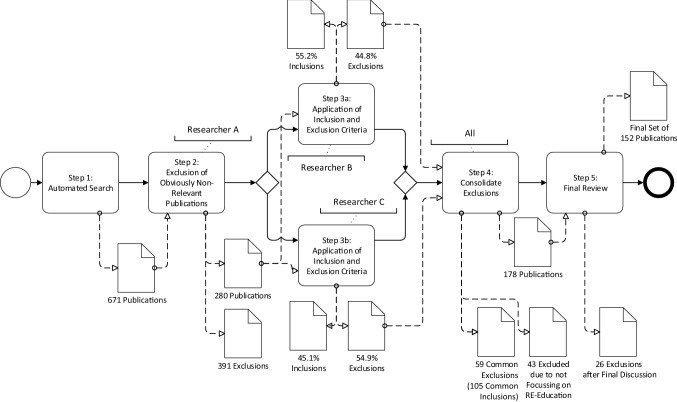
Study selection process

**Fig. 2:**
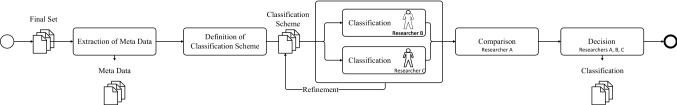
Data extraction process

Figure 1 shows the process of step-wise exclusion of studies to derive the final set of included studies. The studies were selected and excluded at different stages.

Step 1: Automated search using the search string resulted in 671 publications to be considered.
Step 2: In the first round of exclusion, 391 papers were excluded by Researcher A as they were very obviously of no relevance to the field, were non-peer-reviewed publications, not in English, or for other obvious violations of the exclusion criteria. This left 280 inclusion candidates.
Step 3: In a second round, two other researchers applied the inclusion and exclusion criteria on the remaining 280 papers individually. The separate application of inclusion and exclusion criteria was chosen to improve the quality of the paper selection process. In cases of differences agreement was reached in later discussions. In this step, Researcher B included 55.2% and excluded 44.8% of the inclusion candidates, while Researcher C included 45.1% and excluded 54.9% of the inclusion candidates.
Step 4: In this step, the difference in inclusions and exclusions between Researcher B and C were investigated in detail. We identified 105 common inclusions and 59 common papers where both researchers agreed on exclusion. This yielded an inter-rater agreement of 76.92%, $k=0.5217$ (which is “fair agreement”). As this big difference was surprising, the situation was discussed between all researchers. It was noticed that most differences resulted from papers that were not about RE education in the first place but discussed RE education in the context of SE education or in more general curricula. A close investigation of these papers yielded the understanding that the papers did not provide sufficient detail on the particular aspects of RE education to be included in the study. In total, we excluded 43 REE-related papers that met this criterion. Consequently, after Step 4, 493 papers from the original 671 were excluded, yielding 178 papers as inclusion candidates. Of these, 36 papers (i.e., about 5.4% of the original 671 papers) remained undecided for a last step of conflict resolution.
Step 5: In the final step, Researcher A investigated the undecided 36 papers, proposed a solution for inclusion and exclusion, and the final decision was reached by discussion among all three researchers. From the remaining 36 undecided papers, ten were included and 26 were excluded.

In summary, we investigated 671 papers, from which we excluded 519 papers. Resulting in the final set of 152 included publications.^2^

### Data extraction

The data extraction process is illustrated in Fig. 2. To answer research questions RQ1-6, we extracted each paper’s meta-data from Scopus. For RQ7-9, each included paper was read carefully by three different researchers to extract data pertinent to the research questions. For RQ10, we grouped selected studies into common themes for synthesis. We used word-tags pertaining to the content of a study (e.g., “industry-centric,” “motivation,” or “completeness”) and discussed our findings. Where there was disagreement between any two researchers, a third researcher evaluated the paper. The final classification was reached through discussions among all three researchers.

### Quality assessment

Recently, some SLRs assess the quality of included studies (e.g., [135]), but these assessments lack a common standard. For example, the application of qualitative quality assessment criteria may be seen as difficult and ambiguous particularly when conducted by researchers of diverse backgrounds (e.g., [97]), and may, therefore, result in the erroneous exclusion of study results from synthesis. In addition, as is the case with our search, SLRs do not require primary data (i.e., papers) to have been published with sufficient transparency and quality for application of further empirical methods. Thus, we follow a commonly suggested quantitative approach to quality assessment by only including publications that have been peer-reviewed. Hence, we elected to have the quality assessment criteria be reflected in the inclusion and exclusion criteria outlined in Table 2, instead of conducting an additional subjective quality assessment.

**Table 3:** Measurements and classifications for each research question (see Table 1 for full descriptions)

No.	Short title	Measurement/classification scheme
RQ1	Most active researchers	No. of publications per unique researcher
RQ2	Author networks	Graphical analysis of co-authorships cycles
RQ3	Top venues	No. of publications per unique venue (i.e., conference/workshop series or journal)
RQ4	Papers per year	No. of publications per year
RQ5	Top cited publications	Graphical analysis of citation cycles, i.e., which papers on requirements engineering education are commonly cited by other papers on requirements engineering education
RQ6	Research methods	Classification according to Wieringa et al. [194], see Table 4
RQ7	Contributions	Classification according to Petersen et al. [140], see Table 5
RQ8	Keywords	No. of unique author keywords. Aggregated in case of different spellings, singular vs plural, etc.
RQ9	Learners	Audience of the proposed teaching intervention. Classification: university students (either undergraduate students, graduate students, or not further specified by the paper), industry professionals, school students, unknown
RQ10	Educational approaches and learning subjects	Categorization according to the paper author’s description

**Table 4:** Classification of research methods (RQ6), provided by [194]

Type of research method	Description
Evaluation research	Examines a problem or an implementation of a technique in practice
Proposal of a solution	Proposes a solution technique and demonstrates why it is relevant without offering a sound validation
Validation research	Investigates a proposed solution through a sound validation method such as experiment, for example
Philosophical papers	Suggest a new outlook on something
Opinion papers	Report the personal opinions of the authors
Personal experience papers	Report the personal experience of the authors

**Table 5:** Classification of research contribution (RQ7), adapted from [140]

Type of contribution	Description
Metric	The paper proposes a “defined measurement method and the measurement scale” [77] to allow “quantitative measure[s] of the degree to which a system, component, or process possesses a given attribute” [82]
Tool	The paper proposes a “software product that provides support for software and system life cycle processes” [76]
Model	The paper proposes a “representation of a real” world process, device, or concept” [vocabulary], a “representation of something that suppresses certain aspects of the modeled subject” [72], a “semantically closed abstraction of a system or a complete description of a system from a particular perspective” [82], or a “system of postulates, value declarations and inference rules presented as a description of a state of affairs (universe of discourse)” [78]
Method	The paper proposes an “implementation of an operation” [79] or a “statement of how property values are combined to yield a result” [72]
Process	The paper proposes a “set of interrelated or interacting activities that transforms inputs into outputs” [73], a “predetermined course of events defined by its purpose or by its effect, achieved under given conditions” [81], or a “system of activities, which use resources to transform inputs into outputs [80]
Other	The paper proposes anything not to be placed in the categories above

### Analysis and classification

In this section, we revisit our research questions RQ1-10 and describe how we applied the classification schemas. Table 3 presents an overview of this information.

For RQ6, we used a commonly accepted classification for research methods provided by Wieringa et al. [194]. In doing so, we distinguished between evaluation research, proposal of a solution, validation research, philosophical papers, opinion papers, and personal experience papers (see Table 4). Each paper was mapped to exactly one category. In some cases, the categorization of papers might not be obvious. For example, it can be difficult to distinguish between a *proposal of a solution* and *validation research* because these types differ in terms of completeness and rigor of their evaluation, which may not be fully described. In these cases, the classification was based only on the presentation of the paper. Other evaluation activities that were suggested but not explicitly reported in the paper were not considered. Each paper was then assigned to the category that fit best.

For RQ7, we adapted the scheme proposed by Petersen et al. [140], which has been reused in other mapping studies (e.g., [36, 52]). However, as some papers did not fit well into any of the original categories, we added a category for *other* contributions. Table 5 lists each contribution type. Each paper was assigned to all categories that apply.

### Validity evaluation

In this section, we discuss aspects of validity according to the classification scheme in [141], and the measures taken to mitigate these potential threats.

#### Descriptive validity

Descriptive validity deals with the accurateness and objectivity of an investigation. As threats to descriptive validity are considered more significant in qualitative investigations than in quantitative investigations, we assume that there are no major threats to descriptive validity. We did not use qualitative quality assessment but favored quantitative quality assessment, which supports descriptive validity. Misclassification of papers may have led to threats to descriptive validity for RQ10 in particular. We built our classification to a large extent on existing and accepted classification schemes. We classify papers as intended by the authors (e.g., type of research contribution, educational approach used), which have been substantiated in the peer review process, to avoid threats from misinterpretation. It cannot be completely ruled out that authors and reviewers of one paper might have accepted an erroneous classification. We assume this was rare enough in occurrence to not impact the descriptive validity (i.e., without misrepresenting the field).

#### Theoretical validity

Theoretical validity concerns whether the research questions can be answered with the study setup. A major threat in this category typically stems from selection bias. To avoid this bias, we defined objective inclusion and exclusion criteria and applied them rigorously. Inclusion and exclusion criteria were applied independently by two different researchers, with a third researcher validating the choices. Also, the classification was done by two researchers independently, again with a third conducting quality assurance. In case conflicts in the inclusion/exclusion or classification of a paper arose between any two researchers, another researcher was involved, and the conflict was solved by discussion among all researchers, switching roles between “classifier” and “validator” in order to help each individual maintain an objective point of view.

#### Generalizability

Generalizability of the findings deals with the question, whether the set of papers included into the systematic mapping study are representative and do not miss important aspects. Comparison with previous secondary studies on requirements engineering education (see also Sect. 3.8) indicates that we did not miss a considerable number of relevant primary studies to be included.

#### Interpretive validity

Interpretive validity is concerned with the validity of the conclusions drawn. Hence, researcher biases are a considerable threat. To avoid threats to interpretive validity inclusion and exclusion criteria as well as the classification scheme were not applied by one researcher alone. As outlined above, conflicts were resolved by discussion among at least three researchers that investigated the paper independently. This reduces the threat of researcher bias.

#### Repeatability

To ensure repeatability, we report the search and selection process as well as the inclusion and exclusion criteria in sufficient detail to enable other researchers to verify our work. Moreover, we make our data available online, particularly with regard to RQ10. Additionally, abstaining from applying qualitative exclusion criteria helps improve repeatability. However, it cannot be ruled out that different researchers might have classified some of the papers in some cases into different categories. This is a common threat in systematic mapping studies and systematic literature reviews. Yet, due to the large number of included publications, we are confident that this would not alter the implications of our findings.

### Validity evaluation for goal 3

Regarding goal 3, it is important to ensure that we use common grounds with the study of Ouhbi et al. [135], since this study serves as a baseline for our comparison of how REE has changed over the past decade.

We identified 36 of the 79 studies selected by Ouhbi et al.. Two of these studies were considered the same contribution in our work (i.e., [84]) because the two papers were published in the same venue very close to one another. Of the 43 remaining studies reported by Ouhbi et al., we identified 32 that either did not meet our inclusion criteria (e.g., studies with a primary focus on RE education, rather than education at large using RE methods), or meet our exclusion criteria (most commonly studies that are less than four pages long or dealing with RE for engineering education, see Table 2). One study was unobtainable to us, but reported in Ouhbi et al. (i.e., [139], for which, in fact, we were unable to locate any publication record at all). The remaining seven studies identified by Ouhbi et al. were not identified by us using the process described above. These studies are [8, 23, 42, 91, 96, 199] and [178].

Two of the contributions identified by us are in fact Ouhbi et al.’s work [71, 135]. During Step 4 in Sect. 3.3, we included 50 papers which were published in the time period reported by Ouhbi et al.. Of these, we selected 33 contributions that were not reported by Ouhbi et al.. These papers are [3, 16, 22, 28, 44, 45, 49, 53, 58–60, 62, 83, 85, 98, 115, 116, 118, 122, 127, 133, 134, 143, 151–153, 156, 166, 173–175, 185, 187, 188] and [192]. Thus, we have an agreement of 67.19% with the work of Ouhbi et al., which yields a Cohen’s $\kappa$ of 0.3586 (i.e., *fair agreement*) [26].

When comparing results with Ouhbi et al., search strategy accounts for some of the differences between included studies. We relied on Scopus (as this already covers the established publishers in the field) to search for articles, while Ouhbi et al. used the publishers’ search engines and Google Scholar. We purposefully used a more general search string than Ouhbi et al., as outlined above to investigate what authors believe RE Education shall be concerned with. Additionally, we applied stricter exclusion criteria.

In summary, we found more candidate papers but also excluded more. Like Ouhbi et al., we were interested in metadata about the papers. Yet, they investigated which studies referred to reference curricula, while our investigation focused on educational approaches and learning outcomes regardless of reference curricula, and the change of REE research since Ouhbi et al.’s work. Thus, our work is complementary.

Returning to goal 3, since we have covered sufficient common ground with the work of Ouhbi et al., we can provide valid observations about how the field of REE has evolved over the past decade.

## Results for goal 1

**Table 6:** Top 10 of most active researchers

No.	Author	#
1	**Didar Zowghi**, University of Technology Sydney, Australia	9
2	**Dieter Landes**, Coburg University of Applied Sciences and Arts, Coburg, Germany	7
	**Paola Spoletini**, Kennesaw State University, GA, USA	
4	**Manuel João Ferreira**, University of Minho, Braga, Portugal	6
	**Yvonne Sedelmaier**, Coburg University of Applied Sciences and Arts, Coburg, Germany	
6	**Muneera Bano**, Swinburne University of Technology, Melbourne, Australia	5
	**Marian Daun**, University of Duisburg-Essen, Essen, Germany	
8	**Rodina Ahmad**, University of Malaya, Kuala Lumpur, Malaysia	4
	**Beatrice Donati**, University of Florence, DILEF, Florence, Italy	
	**Gregor Gabrysiak**, Hasso Plattner Institute, Potsdam, Germany	
	**Holger Giese**, Hasso Plattner Institute, Potsdam, Germany	
	**Rafia Naz Memon**, University of Malaya, Kuala Lumpur, Malaysia	
	**Mario Piattini**, University of Castilla-La Mancha, Ciudad Real, Spain	
	**Miguel Romero**, Univ. of Bio-Bio, Chilian, Chile	
	**Siti Salwah Salim**, University of Malaya, Kuala Lumpur, Malaysia	
	**Aurora Vizcaíno**, University of Castilla-La Mancha, Ciudad Real, Spain

**Table 7:** Venues with multiple publications

No.	Venue	#
1	**REET**, international workshop on requirements engineering education and training	32
2	**CSEE&T**, IEEE international conference on software engineering education and training	19
3	**RE**, IEEE international requirements engineering conference	10
4	**ASEE**, annual conference and exposition of the American Society of Engineering Education	6
	**FIE**, IEEE frontiers in education conference	6
6	**iStar(T)**, international iStar (teaching) workshop	5
	**REJ**, Requirements Engineering Journal	5
8	**EDUCON**, IEEE global engineering education conference	4
9	**ICSE**, international conference on software engineering	3
	**ITiCSE**, annual conference on innovation and technology in computer science education	3
11	**CIbSE**, Ibero-American conference on software engineering	2
	**CAEE**, computer applications in engineering education	2
	**ISEC**, integrated stem education conference	2
	**SIGCSE**, ACM technical symposium on computer science education	2

**Fig. 3:**
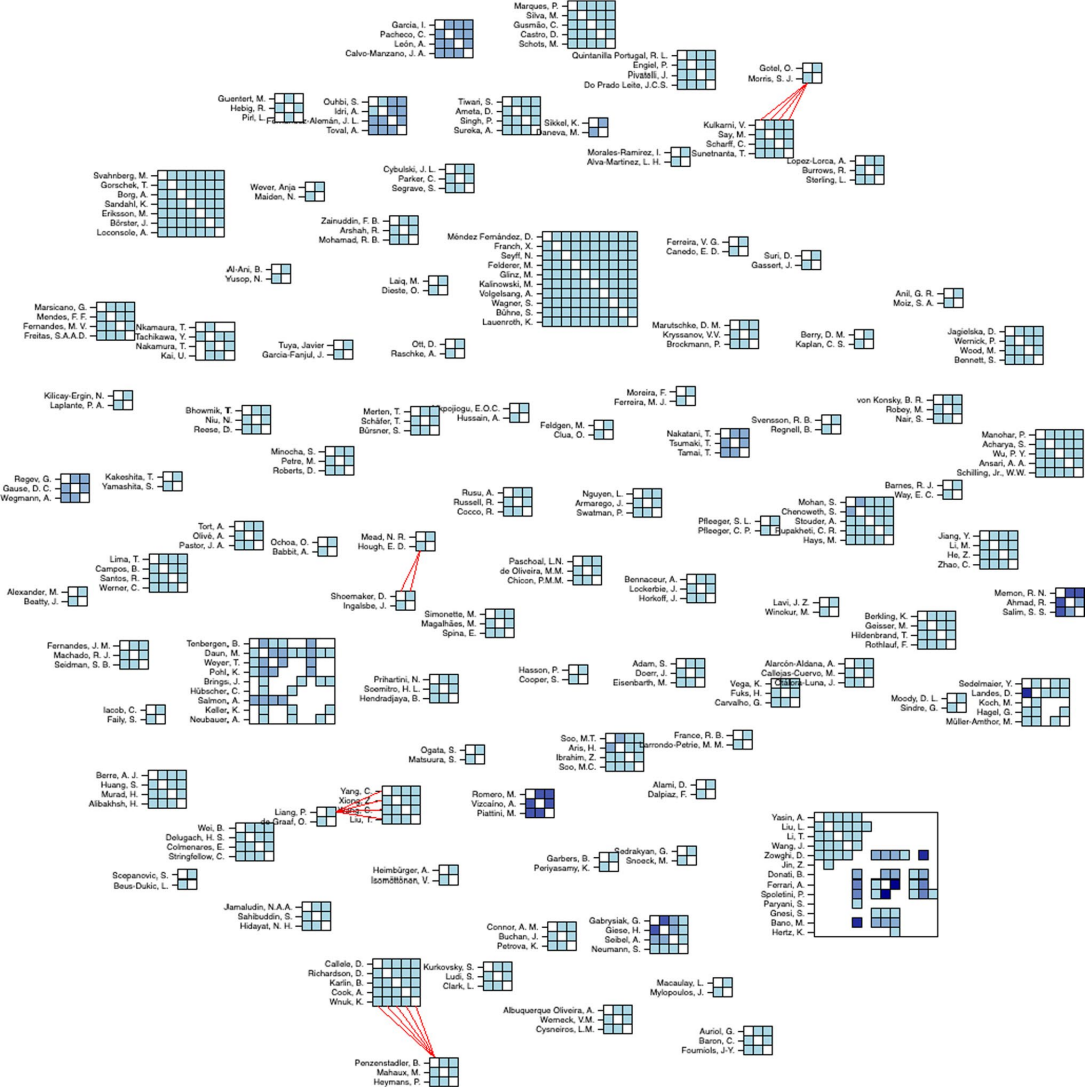
Automatically generated map of author networks. Red lines indicate connections between authors, who are part of two collaboration groups. The darker the hue, the more co-authored papers (Color figure online)

**Fig. 4:**
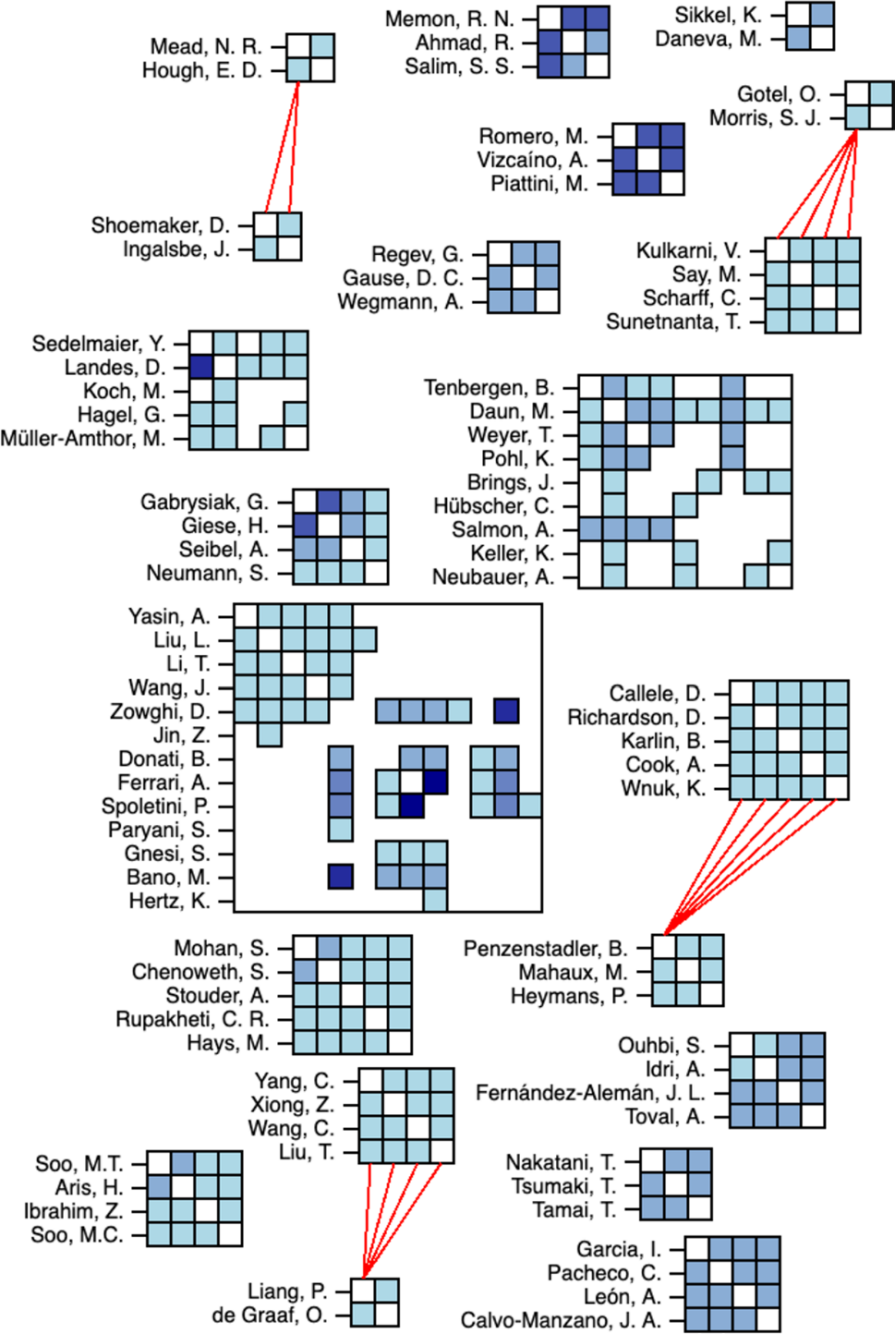
Fragment of author networks only including those with more than one collaboration

**Fig. 5:**
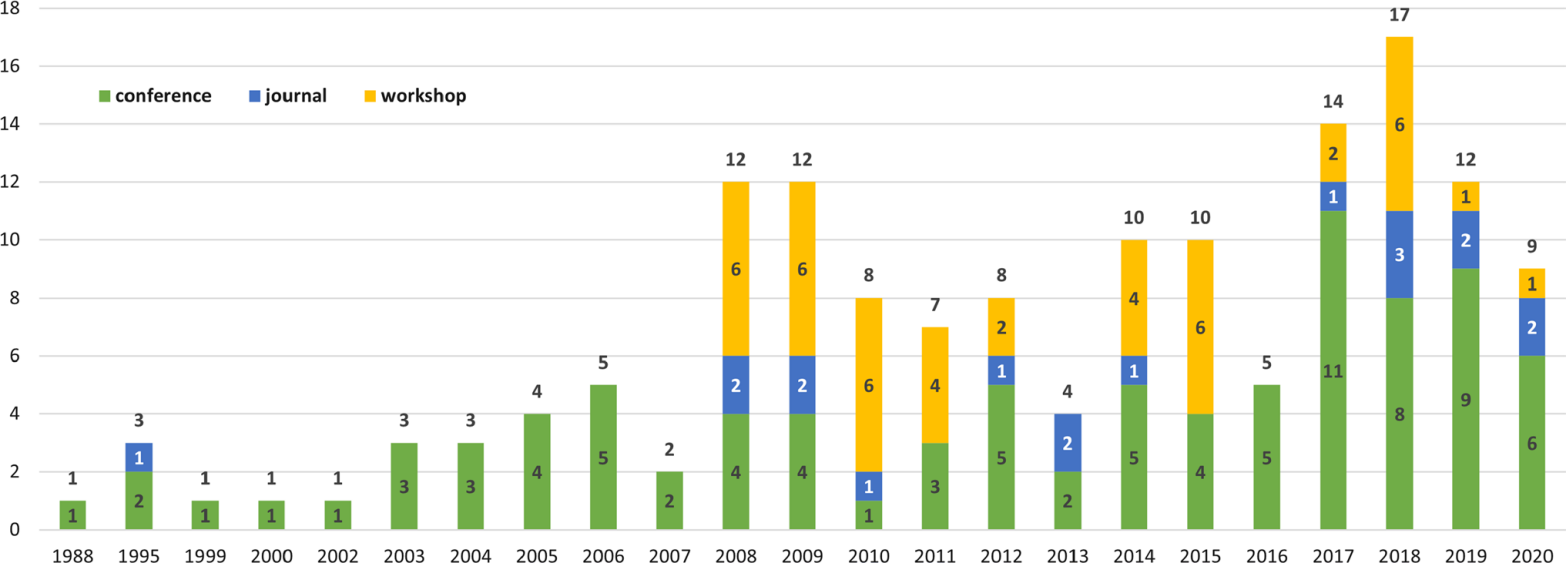
Publications and venues per year. Each bar represents one year, with cumulative counts of publications per year (RQ4) listed at the top of each bar. Bars are sub-divided by type of publication venue (RQ3) to illustrate changes in venue over time

In this section, we present our systematic literature map (goal 1) and explore each research question (RQ1-10) in detail.

### Most active researchers (RQ1)

We begin by exploring who is most involved in REE activities. Table 6 shows the most prolific authors in the area of REE. A total of sixteen authors contributed at least four published papers. Most prolific is Didar Zowghi from the University of Technology Sydney, Australia with nine published papers. As can further be seen, authors regularly involved in REE stem from around the world with a strong focus on Europe. Nine researchers are affiliated with universities from European Union countries: Germany (5), Spain (2), Portugal (1), and Italy (1). Three authors are affiliated with Malaysia, two with Australia, and one with Chile or the United States. Thus, we can see that while 152 articles were selected in our study, the majority of the contributions do not appear to be the primary scientific focus of the publishing scholars (with the exception of the individuals from Table 6), as most authors have fewer than two contributions in this field.

### Research networks (RQ2)

Using study metadata, we automatically generated Fig. 3, which shows the existing networks of authors found in the included studies. This gives a high-level overview of how segmented the efforts are in REE. Rectangles visualize collaborations between individual authors. As can be seen there exists a variety of individual collaborations that are not connected to other authors. Thus, we can assume that the field is rather scattered without collaborations between different author clusters. The coloring indicates the number of collaborations. Most authors participate in only one collaboration (light blue color), the maximum amount of collaborations is four between two authors (dark blue color). To improve readability and further explore existing networks of authors, we isolated networks with more than one collaboration to create a fragment of our map in Fig. 4. Overall, these findings suggest that most selected studies appear to be separate contributions without a systematic continuation of a research direction. A notable exception is the work by Zowghi, Spoletini, Ferreira, and Bano from recent years, which investigates the use of interviews to practice requirements elicitation [13, 40, 41] and inspections [11].

### Top venues (RQ3)

Table 7 shows all venues where multiple papers on REE have been published. We found fourteen venues where researchers regularly publish REE research. Yet, there are only five venues where REE seems to be published on a regular basis (i.e., with more than five total publications). The most established venue for REE is the *International Workshop on Requirements Engineering Education and Training* (REET) with 32 publications. The most established conferences are the *IEEE International Conference on Software Engineering Education and Training* (CSEE&T, 16) and the *IEEE International Requirements Engineering Conference* (RE, 10). The most established journal is *Requirements Engineering* (REJ), yet carries only five publications (of 152 total selected studies). This indicates that so far many early ideas and problem descriptions are elaborated on, with more mature research on REE being rarely addressed in the three most representative venues of requirements engineering research. According to Daneva et al. [29], these are REJ, RE, and the *Working Conference on Requirements Engineering: Foundations for Software Quality* (REFSQ), excluding their workshops. Yet, RE and REJ carry only 15 publications (ca. 9.7% of all 152 selected studies), while REFSQ is not represented in this list at all. Moreover, REJ is merely in sixth place (shared with the iStar workshops). In conclusion, REE research seems to be primarily published in education-related venues that are not specific to requirements engineering as well as the REET workshop. We conclude that there may be a missing connection between non-education research in RE and research specific to RE education.

**Fig. 6:**
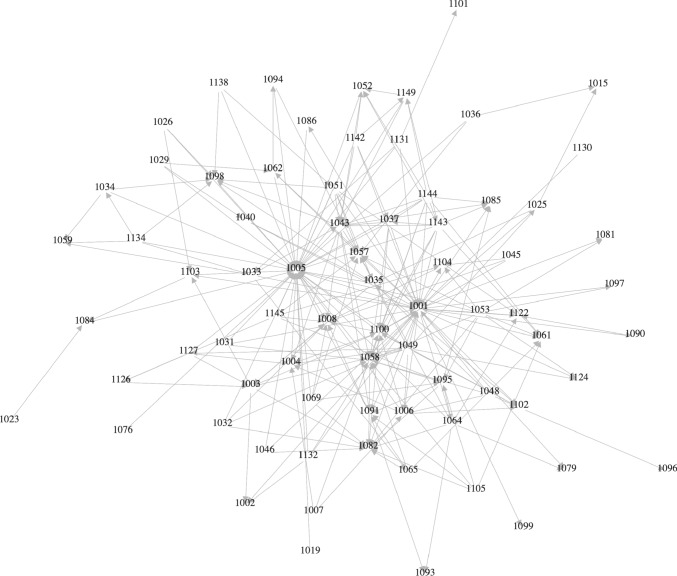
Citation network of articles (dots) being cited by other articles (edges, head pointing to cited article). As can be seen, only six articles are cited four or more times, suggesting no common foundation of RE education literature

### Paper per year (RQ4)

We found an increasing trend of publications over the years. Figure 5 shows the distribution of publications by year and type of venue where a paper was published (see also Sect. 4.3). As can be seen, research on REE started slowly in the beginning with only four conference papers between 1988 and 1998, and one journal article. This was followed by a phase from 1999 to 2007, where papers were regularly published, however in small numbers each year, and only in conferences. Since 2007, REE-related workshops have appeared and are in part responsible for the increase in publications reaching a maximum of seventeen publications in 2018. Thus far, 2018 was the year with the largest number of published journal papers. These findings suggest that REE has gained more and more interest over the years and its importance is shown in still increasing publication numbers. More than half of the publications selected in our work were published *after* the work by Ouhbi et al. [135]. Ouhbi et al.’s work was conducted in 2012 (almost 10 years ago), which coincided with the beginning of a four-year hiatus of REET. The eighth installment of REET was in 2013 and ninth and tenth installments were in 2018 and 2020, respectively. This in turn coincides with a period of slightly decreased frequency of workshop contributions and contributions at the three top venues for RE-specific research [29, 179]: the REFSQ conference, the RE conference, and the Requirements Engineering journal (see also Sect. 4.3).

### Top cited publications (RQ5)

We generated a citation network to analyze citation cycles. Figure 6 shows an excerpt of the citation network, i.e., the set of papers that cite other papers from all included studies. Arrow heads point to papers citing another paper (i.e., can be thought of as an “import” relationship). First, it can be seen that only about a third of all selected studies cite any papers within our set of 152 selected papers at all; the two-thirds of papers not citing any other papers have been omitted from Fig. 6. Second, no paper is cited more than four times (see outgoing arrows in Fig. 6). Most papers cite merely one or two other papers and only five papers cites at least as many other papers (see ID 1001, 1005, 1058, 1008, and 1043 in Fig. 6). These are typically review papers. For example, the paper with the ID 1001 is the review paper by Ouhbi et al. [135]. Thus, we can conclude that no considerable citation cycles do exist. This means that neither is there as standard reference for REE accepted by the community. Thus, we found that most (i.e., at least two-thirds of our selected studies) REE research happens in “a vacuum,” without relying heavily on other findings in the field.

### Research methods (RQ6)

We evaluated the papers based on the presented type of research, i.e., its underlying research method. Table 8 (left-hand side) shows the results separated by year. As can be seen the vast majority of papers are either solution proposals or experience reports. In contrast, evaluation research and validation research are only sparsely conducted. This means that while there exists a plethora of approaches aiming at improving REE and a variety of personal experience reports, more thorough empirical investigations of the field either by exploratory evaluation studies or by thoroughly validated solutions are missing. We conclude from this that the maturity of the field must be considered rather low. This is in line with our findings from RQ5, as indications of high overall maturity would be indicated by common, frequently cited references.

**Table 8:** Data for RQ6: research methods and RQ7: contributions by year

RQ6: Research methods							RQ7: Contributions							
Year	Solution proposal	Experience report	Evaluation research	Validation research	Philosophical papers	Opinion papers	Year	Method^a^	Tool	Framework	Process	Model	Metric	Other
1988	1						1988	1				1		
1989							1989							
1990							1990							
1991							1991							
1992							1992							
1993							1993							
1994							1994							
1995	3						1995		1		2	1		
1996							1996							
1997							1997							
1998							1998							
1999	1						1999	1						
2000	1						2000	1						
2001							2001							
2002	1						2002							1
2003	2	1					2003	2			1			
2004	1	1	1				2004	1	1					1
2005	1		2	1			2005		2			1		1
2006	1	3		1			2006	4		1				
2007	1				1		2007	1						1
2008	3	3	3	2	1		2008	2	5	1	1			4
2009	8	2		1		1	2009	6	2	1				4
2010	1	5		2			2010	4	1					4
2011		3	2	1	1		2011	2	2				1	4
2012	3	1	3	1			2012	4	1	1	1			3
2013	2	1		1			2013	1	1		1	1	1	2
2014	2	3		3	1		2014	3	3		1	1		3
2015	3	5		1	2		2015	5						5
2016	3	1			1		2016	3	1	1				
2017	8	3	2	1			2017	6	2	1			1	6
2018	10	3	1		2	1	2018	6	3	2		1	1	8
2019	7	3	2				2019	8	4		1			2
2020	5	2	2				2020	2	4	3				1
Total	68	40	18	15	9	2	Total	63	33	11	8	6	4	50

^a^Method includes methods, techniques, and approaches

### Contributions (RQ7)

**Fig. 7:**
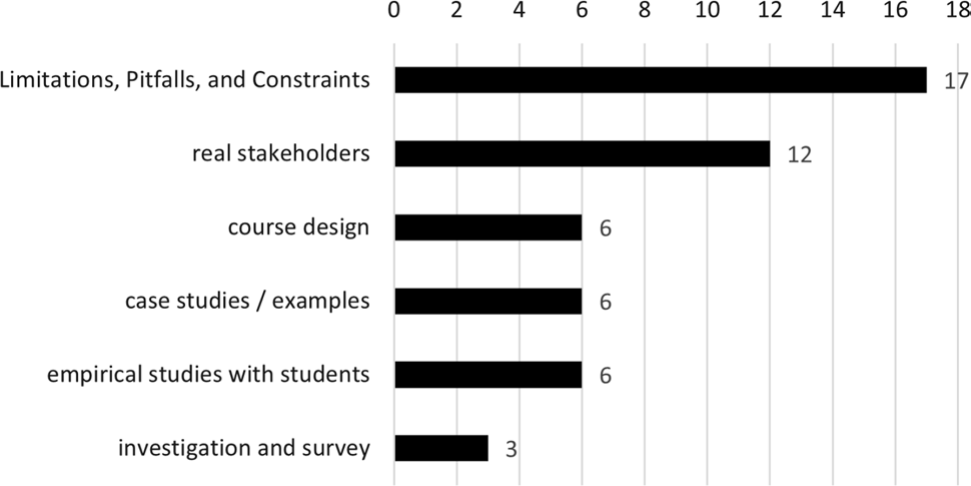
“Other” contributions from Table 8

For the contributions of the included studies, we mapped the publications according to the classification scheme proposed by Petersen et al. [140]. Table 8 (right-hand side) shows the results separated by year. Most publications propose a method, followed by tools to be introduced in REE. This is in line with findings by Malik and Zafar [105] (see also Sect. 2.2). In addition, we found a large number of papers classified as *“other”*. These mostly result from the large number of experience reports, which typically do not propose any kind of contribution in the sense of the categories in [140]. Nevertheless, we classified them according to their common theme, as shown in Fig. 7. As can be seen, many papers classified as “other” in Table 8 report on limitations, pitfalls, or constraints, yet without specifying concrete solutions (17 in total). A total of 12 papers are concerned with involving real or realistic stakeholders (e.g., through role playing), while six papers propose a course design (without explicitly proposing it as a solution to a specific problem). Six more papers propose education research case studies and/or examples (often conflating the two terms), while again six studies report on empirical studies with students, surveys, or other types of investigations, yet without validating or evaluating a proposed solution (see Table 8). We infer from this that unlike non-education fields of software engineering, REE is fairly diverse, yet centers around proposing specific methods or approaches, or involving specific tools. This is in-line with our finding that most contributions are solution proposals (see RQ6). Although this further indicates low maturity of the field, it also means that a diverse set of contributions and solution avenues exist to teach RE, thereby suggesting a rich (albeit unsystematic) “toolbox” of educational approaches.

### Keywords (RQ8)

Table 9 shows the ten most frequently used keywords. As can be seen most keywords are basic terms. Beside these keywords, other keywords are used five times or less often. Thus, this indicates that—beside the topic of requirements elicitation—there seems to be no specific area of interest in requirements engineering that education research particularly focuses on. The frequent use of the term “requirements elicitation” indicates that for this specific area of RE there may be a particular interest in how to teach this topic. Yet, other areas of RE may not receive as much attention. This may make it difficult for educators interested in the field to find a solution to an instructional problem they are faced with, without being intimately familiar with the many solution proposals that exist in the field (see RQ6 and RQ7).

### Learners (RQ9)

**Fig. 8:**
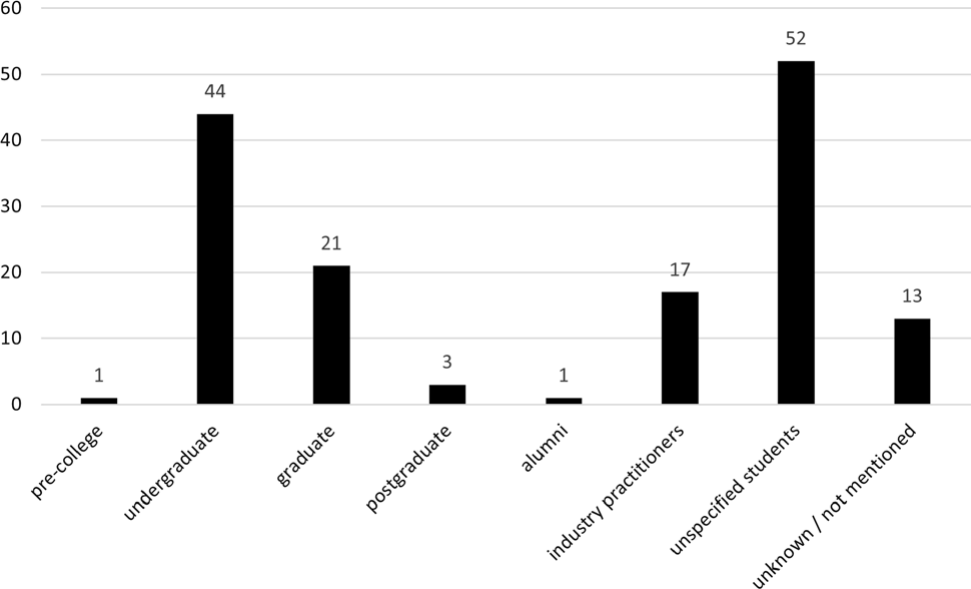
Type of learners addressed

Figure 8 shows the distribution of the emphasized audience of teaching approaches as stated by the included publications. The vast majority of papers (120) clearly address university students. Of these, three papers consider postgraduate students, 21 focus on graduate students, 44 on undergraduates, and 52 do not further specify the level of the learner. Only 17 papers address teaching industry professionals. Thirteen papers omit the audience (“not mentioned” in Fig. 8) or generically speak of “students” (“unknown” in Fig. 8). We assume some of these address university education and find this sufficiently obvious that authors do not deem it important to specify this further. One paper places emphasis on RE education at the high-school level and another one investigates RE knowledge in alumni. This seems to show that Shaw’s “aspiration” [162] was in part answered, as a substantial number of approaches target aspiring software engineers in very early stages (i.e., at the undergraduate level) to instruct role-specific skills related to requirements engineering. Yet, by comparison, industry training is currently not a key focus in REE research.

**Table 9:** Top 10 of most frequent keywords

No.	Keyword	Papers
1	Requirement(s)	55
2	Requirements Engineering	54
3	Learning	45
4	Education	36
5	Requirements Engineering Education	33
6	Software	25
	Teaching	
8	Software Engineering	22
	Analysis	
10	(Requirements) Elicitation	19

**Fig. 9:**
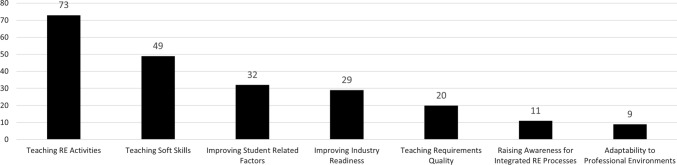
Topics of interest

### Learning outcomes (RQ10)

To gain insights into what the included studies propose or investigate—and thus on the question what the current state of research in REE deals with from a content-related point of view—we identified broad recurring themes. Figure 9 shows these themes and their frequency.

- *Teaching requirements engineering activities. *Most papers (i.e., 73) are concerned with teaching different RE activities. Recurring activities are elicitation, negotiation, specification, requirements validation, management, and modeling. In addition, specific activities as safety analyses or requirements tracing are concern of some publications.
- *Teaching soft skills.* Forty-nine included studies focus on teaching soft skills when teaching RE. Targeted soft skills are typically closely related to the work profile of a requirements engineer. Papers commonly focus on communication skills, teamwork and collaboration skills, conflict resolution skills, interviewing techniques, or technical writing.
- *Improving student-related factors.* In this category, 32 papers aim at improving the learning of students by increasing student motivation, enthusiasm for the subject matter, coping with overwhelmed students, or aim to improve students’ ability to explore problems and deal with solution uncertainty.
- *Improving industry readiness.* A total of 29 publications aim at improving industry readiness of the students to cope with real RE problems. This is typically done by involving real stakeholders in a course, using or investigating real requirements specifications, or applying industry-realistic examples in the classroom.
- *Teaching requirements quality.* In total 20 papers, focus on improving students’ sensitivity to high-quality requirements. Requirements quality properties mainly include consistency and correctness of requirements and requirements specification documents, but also ambiguity, and completeness.
- *Raising awareness for integrated RE processes. *Although these eleven papers were included as they place particular emphasis on teaching RE, their focus lies on doing so as part of a broader development context, e.g., dealing with real customers’ needs.
- *Adaptability to professional environments.* Nine papers propose specific educational settings to foster professional RE skills. For instance, this includes distributed global settings to mimic spatial separation of teams or teaching computer science students together with students from other disciplines to raise awareness of multidisciplinary issues.

This list shows that teaching requirements engineering activities is only part of what REE is concerned with, as about half of the papers deal with non-core requirements engineering theory.

In summary, the results presented in this section constitute our systematic map, which addresses goal 1. It is notable that since Mary Shaw’s aspiration (i.e., to include more role-specific undergraduate software engineering education, see [162]) has been answered by the REE community. A vast plethora of approaches have been proposed, especially since 2012 and beyond. Yet, the field suffers from low overall maturity. Most research appears to be solution proposals, without suggesting a continuing research avenue, and without producing a core area of expertise, neither surrounding scholars, nor surrounding methods, nor surrounding specific contributions. Nevertheless, we found that successful requirements engineering instruction encompasses more than theory, i.e., student factors and soft skills, as well as industry-readiness. Therein lie core themes in the papers we have discussed. In the next section, we address goal 2 and discuss the most significant trends pertaining to learning outcomes, as well as the educational approaches to achieve them.

## Results for goal 2

Next, we explore goal 2 of this paper, which investigates the current practices regarding pedagogical techniques and the learning outcomes they seek to achieve. We initially hoped to distill these practices based on data from validated approaches. However, as can be seen by the results of RQ6 and RQ7 (see Sects. 4.6 and 4.7, respectively), research contributions are overwhelmingly solution proposals or experience reports, with only 21.7% being evaluation or validation research. While several proposed solutions provide at least minimal quantitative or qualitative evidence as to their efficacy, a systematic replication and investigation of their pedagogical benefits is (unsurprisingly [25, 163]) largely missing.

Nevertheless, as can be seen by the results from RQ10 (see Sect. 4.10), there are some clear and promising trends. These trends can be summarized into the following topics:

- Authenticity and industry-readiness
- Teaching RE activities and requirements quality
- Student factors and soft skill development

**Fig. 10:**
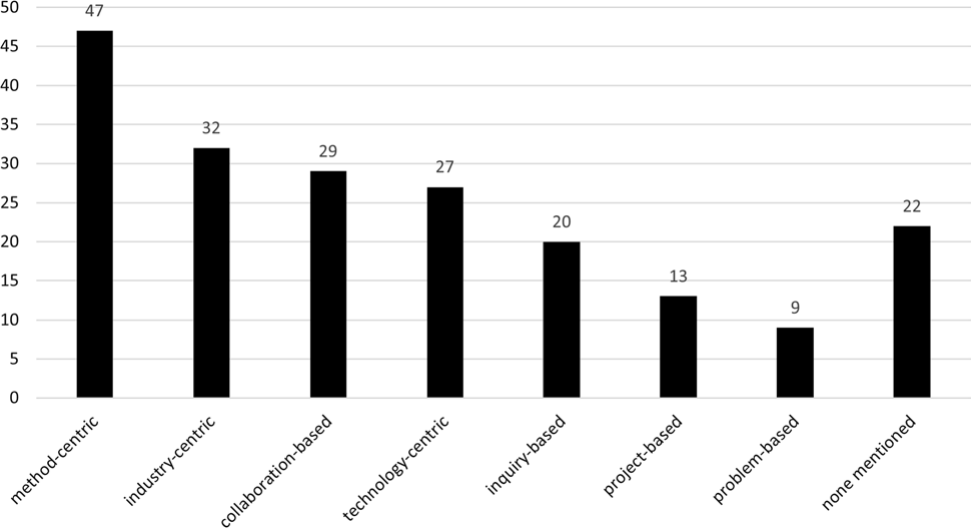
Type of educational approach

To give further context to our discussion of learning outcomes, we tagged the papers in our mapping based on their educational approach, as explained in Sect. 3.4 shown in Fig. 10.

**Fig. 11:**
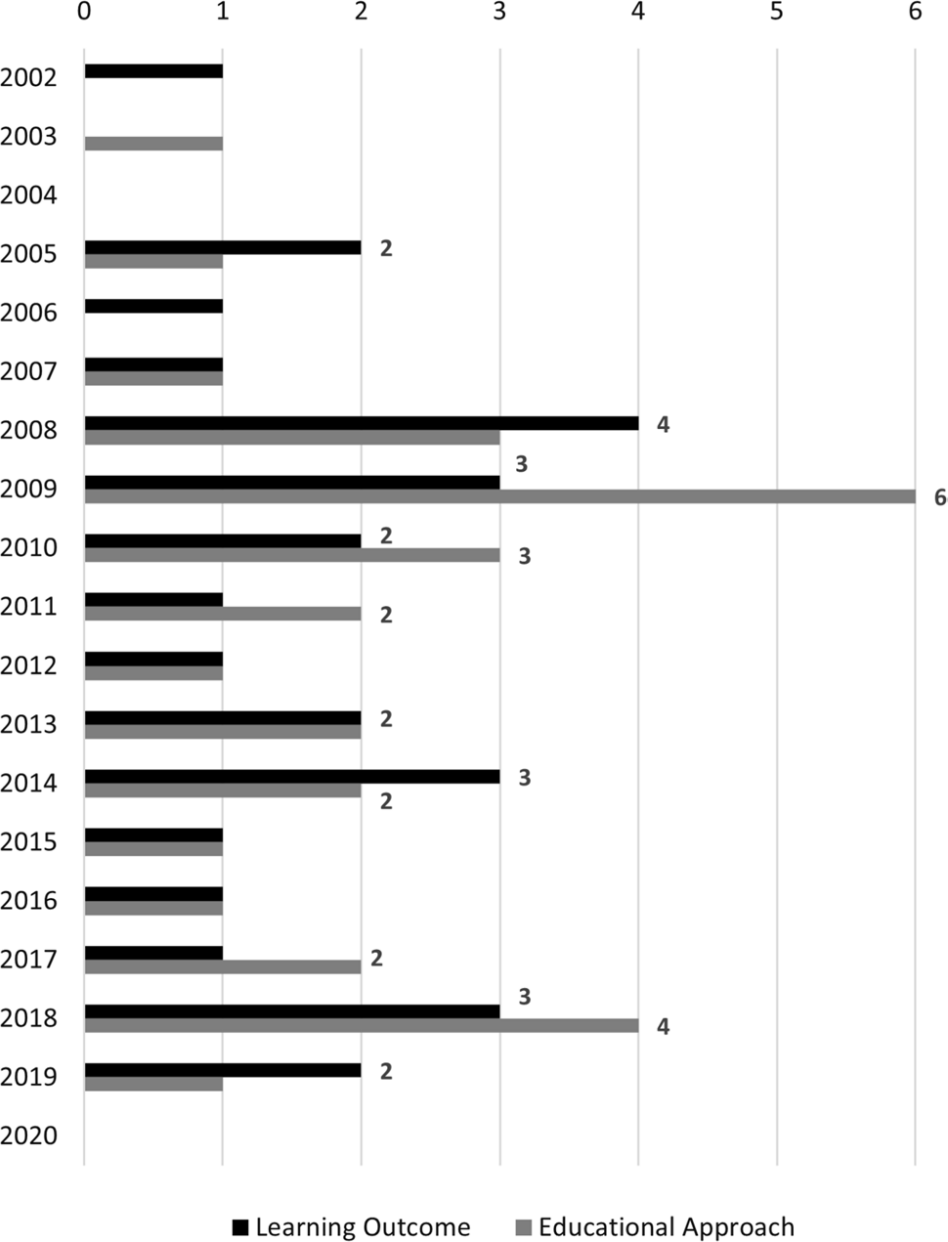
Publications per year proposing an industry-centric learning outcomes and educational approaches

**Fig. 12:**
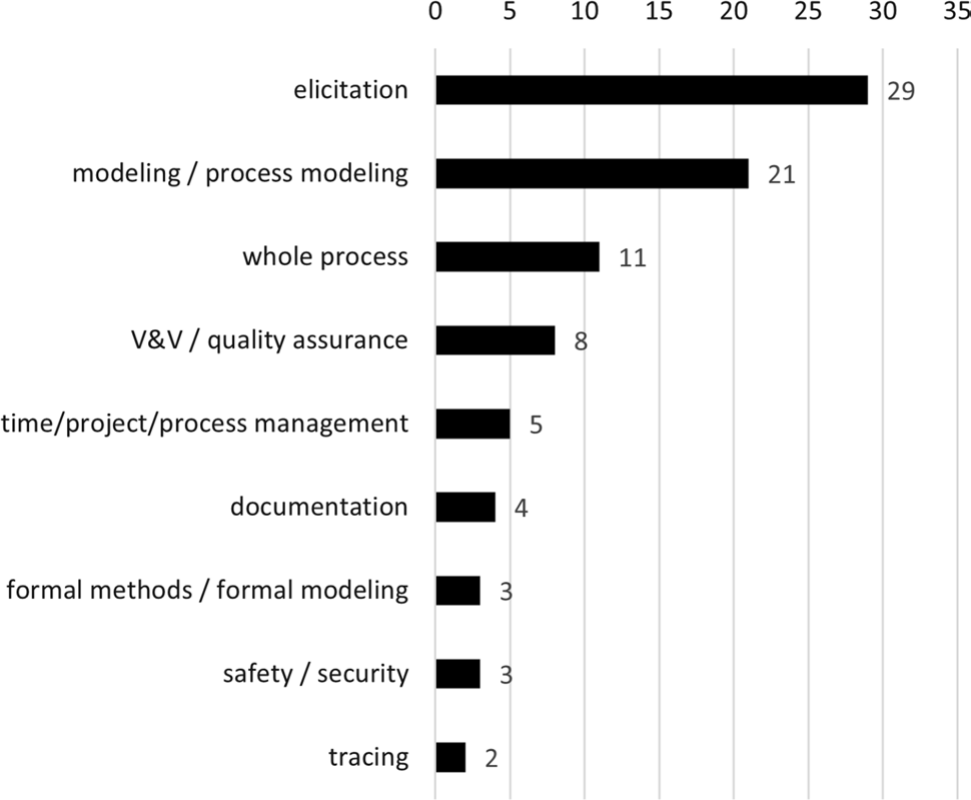
Studies explicitly instructing RE activities

### Authenticity and industry-readiness

The first trend that we observed is the prominence of work that focuses on industry-readiness and giving students an authentic RE experience. We found that 32 papers (see Fig. 10) used an industry-centric educational approach (e.g., by involving external stakeholders from real companies), and 29 papers (see Fig. 9) explicitly mention industry-readiness as a learning outcome. Figure 11 shows these studies over time. From this timeline, we can see that this research focus is comparatively young, as half of these approaches have been published in the past 10 years alone.

Further, this trend suggests that the community is moving away from instructor-centric approaches, which focus on rote memorization of theory and individual high-stakes problem solving. Instead, nearly two-thirds (61.84%) of the studies shown in Fig. 10 propose a non-instructor-centric approach to instruct RE. Note that in Fig. 10, approaches could pertain to more than one category. Nevertheless, we found 94 individual studies. These include the 32 aforementioned industry-centric approaches, as well as student collaboration (29 studies), project-based (13 studies), problem-based (9 studies), and other inquiry-based paradigms (20 studies (e.g., games [5, 50] or case studies [110, 182]). Of the 32 industry-centric studies, seven studies do so by fostering student collaboration (i.e., [27, 38, 123, 125, 167, 177, 180]), four do so through project-based instruction (i.e., [9, 16, 32, 180]), and four through some other form of inquiry-based instruction (i.e., [27, 53, 168, 177]). Within these 32 studies, two paradigms around stakeholder involvement can be differentiated: on one hand, approaches involve external stakeholders in realistic projects (e.g., [48, 61, 103, 137, 138]); while on the other hand, approaches involve the instructor (or some other non-industry representative, e.g., [47, 177]) to engage in role-playing to create an industry-realistic project experience (e.g., [98, 125, 130, 168, 176]). In both of these paradigms, stakeholders serve as a partner to help students with requirements activities to some degree (see Sect. 5.3).

However, achieving industry authenticity is not necessarily done by involving real or mimicked stakeholders alone. Other approaches include using industry-realistic case examples (e.g., [30–32]) or using geographically distributed teams working on the same project (e.g., [9, 16, 149, 152, 198]). These approaches are interesting because they address soft skills in addition to industry-readiness (also see Sect. 5.3).

Evidence on the effectiveness of improving industry-authenticity relies on experience reports (e.g., [98, 138, 171]). Quantitative data are mostly available by means of students’ course evaluations (see [147]), or exam results (see [32]). Perhaps this is because student evaluations, assignment sheets, and exams are the typical means of assessment of student performance; however, another way of assessing learning outcomes is to measure student performance against industry needs, such as through a graduate alumni surveys of preparedness. This was done in [184], where researchers found that perceived usefulness of instructed documentation formats (e.g., use cases or glossaries) seem to increase with graduates’ work service and project experience.

In summary, we consider it a positive development that educational approaches have taken a keen focus on improving students’ industry-readiness and are moving away from rote memorization in favor of formative learning. However, many of these approaches aim at doing so without consideration of industry needs. Few approaches report on providing requirements engineering training to practitioners, with the notable exception of Morales-Ramirez et. al’s work in [123]. For both, more studies providing evidence are desirable.

**Table 10:** Techniques used by “elicitation” studies in Fig. 12

Technique	Count	Studies
Interviews	19	[11–13, 28, 35, 40, 41, 48, 51, 69, 95, 101, 136, 150, 156, 174, 176, 177, 198]
Workshops	1	[102]
Group techniques	1	[53]
Diagram review	1	[86]
Various	1	[50]
Unspecified	6	[46, 121, 126, 149, 157, 161]

**Table 11:** Instructional approaches used by “elicitation” studies in Fig. 12

Approach	Count	Studies
Role playing	9	[11–13, 40, 41, 69, 176, 177, 198]
Real stakeholders	4	[46, 48, 53, 174]
Games	4	[50, 51, 101, 156]
Tool support	4	[86, 95, 136, 150]
Problem-based learning	2	[121, 157]
Collaboration	2	[102, 149]
Simulation	1	[28]
Competence-oriented	1	[161]
Didactics		
Unspecified	2	[35, 126]

### Teaching RE activities and requirements quality

Next, we investigate trends in selecting topics to include in RE training. About half of the investigated studies focus on specific RE activities (73 studies in total, see Fig. 9). We visualize our selected categories of this breakdown in Fig. 12. Among the most common are elicitation (39.72% of studies, e.g., [53, 69, 86, 102, 150, 174, 176]), modeling (28.77% of studies, which includes “modeling syntax” [17, 34, 66] and “process modeling” [107, 159]). Eleven studies (15.07%) explicitly aim to instruct the whole RE process, while validation, verification, or quality assurance are a topic in only eight studies (10.96%, e.g., [41, 69]), and management in only five studies (6.85%, including “time management,” “project management,” or “process management,” i.e., [14, 38, 100, 114, 121]). Surprisingly rarely do studies investigate more rigorous RE activities. For example, we found only three studies that look at security requirements engineering (from a process perspective, not quality perspective, i.e., [110, 111, 143]), three studies investigating formal methods (i.e., [44, 188, 191]), and two studies on requirements tracing (i.e., [19, 116]). Safety requirements engineering was merely the elementary instructional focus of a single study (i.e., [180]).

Looking closer at the most investigated RE activity, elicitation, the vast majority of included studies (i.e., 19 out of 29) do so by means of using interviews as the predominant technique (see Table 10). Of the remaining ten papers, six studies did not specifically emphasize any particular elicitation technique, while three used specific technique (e.g., workshops) and one used *various* elicitation techniques including interviews.

Additionally, we considered which instructional approaches were used in elicitation activities. As shown in Table 11, papers that used role playing were the most predominant approach. Other approaches included using real stakeholders, games, and tools. The contribution by Sedelmaier and Landes [161] was particularly noteworthy in this respect, not only because it is one of the few studies that employ a specific pedagogical paradigm (i.e., “competence-oriented didactics” in Table 11). While two papers did not specify any approach, we did not find any papers that studied the use of competitor or market analyses.

It is also noteworthy that some requirements engineering activities that could be considered essential (e.g., negotiation or prioritization) are not specifically targeted by RE education at all, as shown in Fig. 12. While these activities may be subsumed in those studies targeting the “whole process,” the respective authors did not explicitly list all activities they included.

**Fig. 13:**
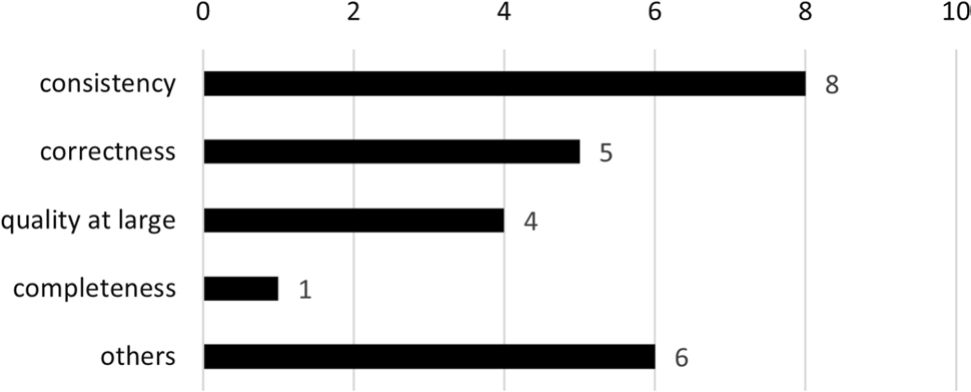
Studies explicitly instructing requirements quality

We observed a mismatch between teaching requirements quality properties (i.e., completeness, consistency, and traceability) and work advocating for a project-based learning environment. We found a total of 20 studies that explicitly mention teaching students to be sensitive to requirements quality. Of these, only two also pertain to those included in Sect. 5.1 (i.e., [58, 180]). Figure 13 shows the breakdown of quality requirements publications, where studies may target more than one quality. Of the remaining 18 studies (see Fig. 13), the predominant focus is on requirements “consistency” (i.e., [49, 62, 69, 166, 174, 188, 190, 191]). “Correctness” is targeted by five studies (i.e., [7, 49, 58, 87, 188]); however in doing so, studies often conflate formal provability of requirements and the sense of adequacy with regard to stakeholder needs (see [55, 57] for a discussion of the difference). Only one included study explicitly mentions “adequacy,” but this is specifically in the context of security requirements [131]. Similarly, “completeness” is only explicitly targeted by Westphal in [191], albeit in the context of formal modeling of requirements. Four studies do not limit the educational focus on individual qualities, but rather mention “quality as a whole.” These studies are [11, 41, 53] and [59]. A total of six studies implicitly target requirements quality (“others” in Fig. 13). While they explicitly state the need to instruct sensitivity to high-quality requirements, the educational approach therein is not specifically targeted to requirements artifacts, but rather activities to improve quality in requirements. In this sense, “complexity” or “abstraction” are mentioned by three studies (i.e., [37, 93, 114]). The remaining studies each mention one quality property: traceability [60], ambiguity [174], and understandability [175].

As outlined in Sect. 4.6, many of the studies we surveyed are “solution proposals” (see Table 8). Of these, most advocate for project-centric collaborative approaches and a minority advocate for instructor-centric, theory-heavy instruction. This is consistent with our earlier finding (see Sect. 5.1) that most approaches advocating industry-readiness do so in a project-based setting, requiring students to experience the whole RE process, from elicitation to documentation, to management. However, only ten of 73 studies mention a specific RE activity in an industry-realistic setting as opposed to targeting the whole RE process or not mentioning RE activities at all (i.e., [28, 32, 48, 50, 100, 106, 126, 128, 180, 184]).

In summary, we found very little overlap between studies mentioned in Sect. 5.1 with studies aiming to teach RE activities and a focus on requirements quality. This seems to suggest that by increasing industry-readiness comes at the expense of teaching specific RE activities and requirements quality. However, we do not believe this to be the case. Many of the studies mentioned in Sect. 5.1 aim to convey a feeling of the intricacies of the whole RE process to students, not just individual activities. Moreover, it is the whole process experience which highlights issues such as completeness of requirements through elicitation and documentation, adequacy/correctness of requirements through validation and verification, requirements consistency and the like. However, while not ignored, it seems that these intricacies are at best conveyed implicitly. We did not find any study explicitly investigating how industry-readiness may also foster requirements activity proficiency and sensitivity to requirements quality, and recommend this as an area for future research.

### Student factors and soft skill development

**Fig. 14:**
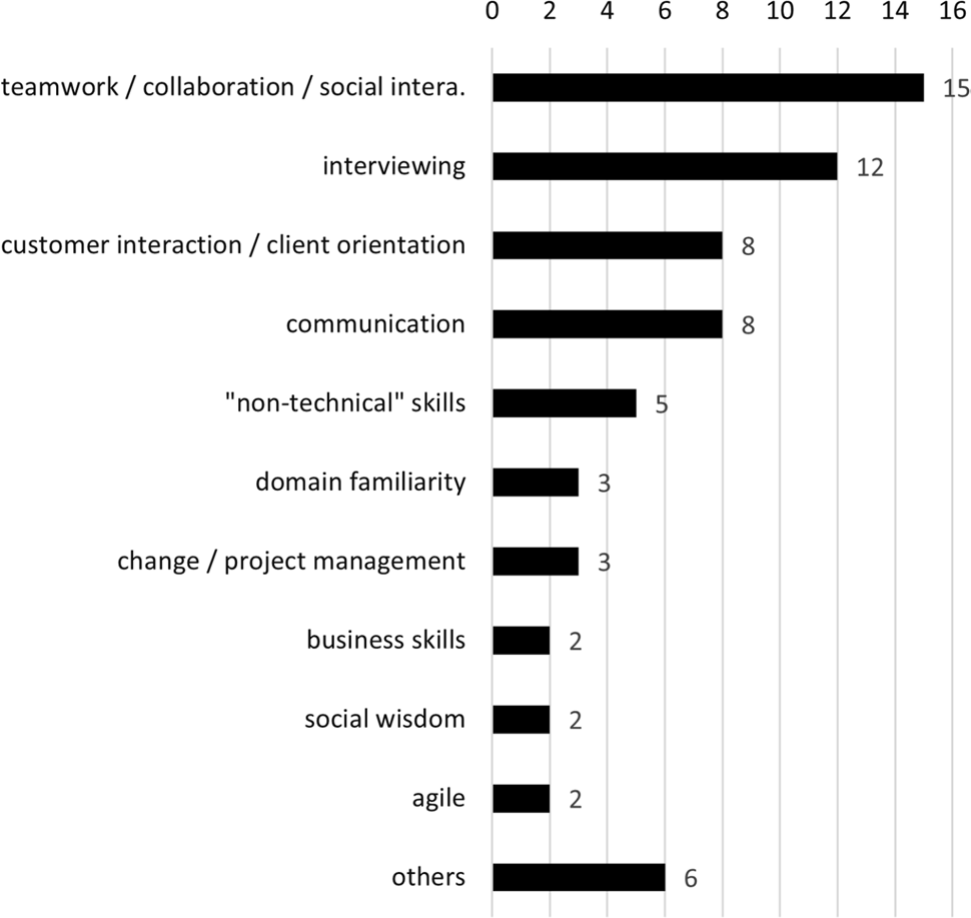
Studies explicitly improving soft skills

A third theme we found in our analysis is that many of the included studies emphasize student factors and soft skill development. This means that the focus is on *“how”* to conduct requirements activities effectively, thereby increasing soft-skills such as communication (as in [177]) or customer-orientation (as in [174]) rather than solely teaching *“that”* requirements elicitation is necessary. We tagged the publications emphasizing soft skills and visualize our results in Fig. 14. The most frequently addressed soft skills are teamwork, collaboration, or social interaction (30.61% of studies pertaining to soft skills, e.g., [16, 31, 32, 66, 118, 138, 147, 153, 180, 187]), interviewing skills (24.49%, e.g., [12, 13, 28, 35, 156, 176, 198]), customer interaction or client-orientation (16.33%, e.g., [69, 118, 125, 127, 157, 174]), and communication (also 16.33% of soft skill studies, e.g., [27, 149, 152, 153, 159, 168, 177]). Only two studies focused on agile development as a soft skill (namely, [67, 102]); however, most studies focusing on collaboration and communication applied a project-centric and industry-realistic (see Sect. 5.1) learning environment in conjunction with agile methods. Like in Sect. 5.2, the overlap to those studies in Sect. 5.1 is fairly low, as only four studies appear to explicitly involve authentic industrial settings to improve students’ soft skills, i.e.,  [28, 67, 118, 180].

However, as introduced above, most of the contributions whose primary focus is on soft skill development do so in a collaborative and/or project-based setting. Of the studies that apply a formative instruction paradigm (i.e., project-based, problem-based, and/or collaboration-based instruction in Fig. 10) and of the 49 studies that aim to improve students’ soft skills (see Fig. 9) as their primary learning outcome, the overlap consists of 19 studies (i.e., 30.65% of formative methods studies from Fig. 10). These studies mainly focus on communication, interviewing, and team collaboration in project-based settings.

The overlap between the same formative instructional approaches from Fig. 10 and studies specifically aiming to improve student factors is much lower. We identified a total of 12 studies that employ a project-, problem-, or collaboration-based instructional method in combination with the explicit aim of improving student factors. These factors include enthusiasm and motivation (e.g.,  [31, 98, 108]), comprehension and understanding (e.g.,  [103, 117, 181]), learning and retention (e.g.,  [16]), and introspection (e.g.,  [43, 94]), which is a 19.35% overlap with formative approaches.

**Fig. 15:**
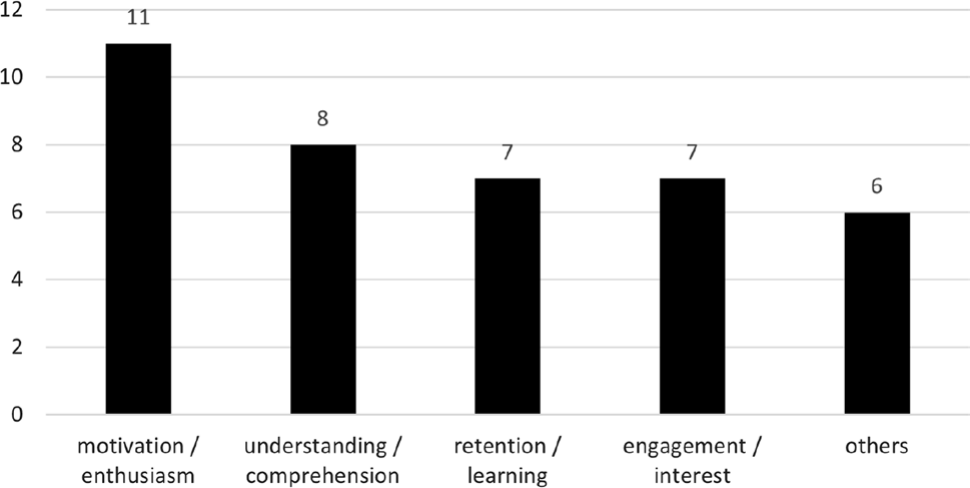
Studies explicitly improving student factors

In total, we found 32 studies that propose a diverse set of pedagogical strategies to improve student factors, which we show in Fig. 15 (note, studies may pertain to more than one student factor). The most commonly addressed student factors are motivation/enthusiasm (11 studies in total i.e.,  [5, 31, 32, 34, 50, 51, 94, 98, 117, 138, 180]), understanding/comprehension (8 studies, i.e., [44, 63, 103, 117, 159, 170, 181, 188]), retention/learning (7 studies, i.e.,  [6, 15, 16, 34, 66, 122, 187]), and engagement/ interest (also 7 studies, i.e.,  [5, 31, 32, 94, 117, 138, 180]). The remaining six studies target a diverse, yet more abstract set of student factors, i.e., “combating students being overwhelmed” [190], “effort and aggravation” [58], “review effectiveness” [134], “acceptance of uncertainty” [14], “process competency” [129], and “introspection” into the validation process (i.e.,  [11, 13, 41], which for the purpose of this discussion, we consider one contribution).

Besides formative and industry-centric approaches as outlined above, studies aiming to improve student factors and soft skills propose a diverse set of strategies to fulfill their aim. In particular, the use of games or gamification (e.g., [5, 6, 34, 50, 99, 156, 170, 187, 196]), engaging case examples (e.g., [3, 9, 30]), or using low-stakes assignments (e.g., [18, 28, 196]) are promising approaches that emerge from the literature.

In summary, while proposals for teaching specific RE activities are separate from improving student factors and soft skills (see Sect. 5.2), we found that student factors and soft skills are a tangential learning outcome of this work. By comparison, industry-authenticity specifically adopts external stakeholders or role playing as an instructional mechanism in order to improve student motivation and enthusiasm (see Sect. 5.1). The REE literature recognizes that soft skills are critical for students’ success in future employment and that student factors are critical in improving student success in requirements engineering. Nevertheless, more work on how to successfully and holistically integrate theory instruction and student success is desirable.

## Results for goal 3

In this section, we address goal 3 by evaluating how REE has changed over the last decade. To accomplish this goal, we compare our findings to those from Ouhbi et al. [71, 135]. In Sect. 3.8, we compared our literature search methodology and results to those from Ouhbi et al. to contextualize our analysis. In the following subsections, we compare to Ouhbi et al.’s “implications and advice for instructors” and what REE research has contributed since the study concluded. Finally, we identify additional gaps in current REE literature and offer our own conclusions.

### How literature addresses Ouhbi et al.’s “implications”

Following a detailed map of the REE field, Ouhbi et al. provide advice to REE instructors in the form of seven implications derived from their selected studies. In this section, we discuss these implications and contrast them with studies published after the period of investigation reported by Ouhbi et al., which allows us to consider the progress in the field since 2012. Furthermore, we expand on these implications with our own observations and recommendations for REE instructors.

#### Combating vague requirements

Ouhbi et al. recommend that instructors teach proper problem scoping in order to avoid vague requirements. The authors assert that certain personality traits improve team performance in this respect. Indeed, such a relationship exists [164, 165, 189] and as we have outlined above, student factors such as comprehension, effort, and enthusiasm are explicitly mentioned learning outcomes in 32 of our 152 selected studies. Eighteen of these studies fall into a time frame *after* Ouhbi et al.’s search completed. While none of these studies mention “vagueness” or “attention to detail” explicitly, several mention “introspection” (e.g., [13]) or “comprehension” (e.g., [117]). Unfortunately, “vagueness” or “level of detail” was not mentioned as a learning outcome in any of our selected studies. We conclude from this that instructors have recognized that student factors are crucial in educating students to become effective requirements engineers, yet student factors alone do not yield effective requirements specifications. We recommend instructors consider pedagogical techniques aimed to increase the level of detail and thereby combat vagueness in requirements specifications.

#### RE tool instruction

With hundreds of RE tools available on the market, Ouhbi et al. make a strong argument for the need to educate students into using these tools effectively. Indeed, 27 of our selected studies deal with tools or advocate using technology to improve learning and instruction. However, the overwhelming majority of these studies propose games (e.g., [5, 50, 51, 70, 156, 170, 187, 196]) or simulation tools (e.g., [10, 150, 154]) to teach RE. They do not outline *how to use* tools during the RE process. A notable exception is [87], where requirements modeling using tools is taught as well as [106], which in part investigates the use of tools to conduct validation and verification of requirements. Our results show that RE tool instruction is still lacking, nearly 10 years after the conclusion of Ouhbi et al.’s survey. A focus here should be on industry-typical tools and tools that are likely to produce a tangible benefit to the RE process, for which current industry needs are unknown and must be assessed (see also Sect. 5.1). Nevertheless, using the right tools during RE also depends on the company-specific tool chains and may therefore be “on the job training,” rather than something that can (or should) be instructed at university level. Again, an industry perspective is required to answer this question.

#### Promote requirements modeling, validation and verification, and prototyping

Our results in Sect. 5.2 show that next to elicitation and RE process instruction, the most commonly addressed RE activities are modeling of any kind as well as quality assurance at large (a total of 29 studies, see Fig. 12). Most of these studies occurred before 2012 (i.e., when Ouhbi et al. concluded) with the exception of [17, 66, 87, 117, 125, 184] and [190]. Ouhbi et al. were correct to point out that more instructional focus was required, as these 29 studies made up a mere 19.1% of all our selected studies (compared to 18.4% for “elicitation” alone). We agree that these activities (i.e., modeling and prototyping) should be promoted during RE instruction. Modeling and requirements validation have proven to be a key asset in the requirements engineer’s toolbox to bridge the gap between non-technical and technical stakeholders. Teaching non-technical skills has thus far mostly taken the form of soft skills (see Sect. 5.3), but even in this regard, the focus is on communication and interviewing due to the strong overlap with studies that focus on “elicitation” (see Fig. 14). Prototyping of requirements specifications has not been emphasized or made a key learning outcome in any of our selected studies. An opportunity here is lost in that students do not benefit from seeing the relationship between “theoretic” requirements specifications and their implementation. While we have reported an activity involving requirements prototyping in one of our selected studies [180], this was only a minor milestone in a RE project, burdened by other constraints in the timeline of the semester. We recommend practitioners to develop approaches such as [108] and incorporate requirements implementation as well.

#### Using industry-realistic projects

As outlined in Sect. 5.1, delivering an authentic, industry realistic educational experience has consistently been a focus of REE literature since roughly 2005 (see Fig. 11). In Ouhbi et al.’s study, the focus was on REE approaches and their relationship to standard curricula, many which require industry-readiness as a student outcome (e.g., [1]). While this is a positive trend in the past, we concur with Ouhbi et al. that this remains an important educational outcome for future work in REE.

#### Promote global software development

Ouhbi et al. emphasize the need for REE approaches to meet the demands placed on software development through a consistent move toward distributed teams. In particular, in light of the recent events (i.e., the COVID-19 pandemic), we agree that video conferencing and distributed teamwork have become necessary skills for students, educators, and industry professionals to master, and will likely shape the landscape of software development for the coming years. Teaching effective RE in such a context may be easier going forward because learners may be accustomed to social distancing and working remotely. Nevertheless, only a minority of our selected studies consider distance learning or geographically separated teams, only one of which was published after 2012 (i.e., [16, 108, 149, 152, 198]). This must be a focus of REE approaches going forward, and these approaches could build off of the experiences from forced distancing during the COVID-19 pandemic.

#### Familiarize students with problem solving

Ouhbi et al. highlight the importance of problem solving skills to become effective requirements engineers and recommend REE literature to take a game-based approach to problem solving. While “problem solving” was only explicitly mentioned in one of our included studies (i.e., [17]) and while games-based instruction or gamification is the focus of several of our selected studies (e.g., [5, 101, 109, 187]), we argue that these approaches are not the only strategies to teach effective problem solving. In fact, peer-learning [27], role-playing [4, 98, 168], fostering analytical thinking [64], and client-orientation [157, 174] have been successfully applied to aspects of RE instruction. Problem solving is at the heart of RE. The key caveat seems to be to create a low-stakes environment, where students can “safely fail” (i.e., explore solution alternatives without grade penalty for being wrong or without threatening project success). Approaches that offer low-stakes learning experiences are quite common, both in individual-centered instruction (e.g., [113, 158]) and collaborative instruction (e.g., [28, 31, 119]). In fact, 24 of our selected studies can be roughly categorized as employing some form of low-stakes problem solving experience; a trend that should continue in the future.

#### Use mobile devices as teaching tools

Ouhbi et al. made an argument to use mobile devices and online tools as a vehicle to teach RE. However, Ouhbi et al. did not articulate in what way REE, in particular, benefits from m-learning or e-learning. When examining our selected studies, only three mention some type of online platform or the use of mobile devices to teach RE (i.e., [88, 124, 144]). We conclude from this that the benefits of m-learning and e-learning to REE may still be largely unexplored, beyond the opportunity to prepare students for the challenges of global software development (see Sect. 6.1.5).

### Gaps in current RE education literature

While industry-readiness, authenticity, and student soft skill development are important and encouraging trends in REE literature, in the following sections, we highlight the areas that have not received sufficient attention.

#### Safety and security requirements

Shockingly few studies (i.e., only three: [110, 111, 143]) deal with security requirements and only one study considers safety requirements explicitly [180]. Since software systems are increasingly entrusted with sensitive information and playing a mission-critical role, it is vital that students are exposed to these considerations at the earliest possible stage during their undergraduate curriculum. Further work is required to understand how to effectively instruct learners on the intricate notions of security requirements and their impact on the system under development. While some studies may incidentally involve safety and security requirements, a systematic educational approach is required.

#### Supply chain risk management and supplier/integrator relation

Most project-based approaches involving real or realistic stakeholders aim to convey the difficulty of managing conflicting requirements. However, these approaches may prime students towards an attitude of “document and forget” [32]. Requirements are rarely seen through to their implementation (see “prototyping” in Sect. 6.1). Moreover, typical software engineering projects emphasize software construction. The current literature largely ignores the need to systematically explore reuse of off-the-shelf components, the need to critically reflect on adopting components (e.g., libraries), or risk involved when adopting possible security-critical technologies. The decision to adopt a technology and risk its successful integration are inherently RE-related and must be systematically assessed on the basis of requirements. At present, students do not achieve this learning outcome with the approaches reported herein.

#### Pedagogy in RE education

Systematic application of pedagogy is largely ignored by contemporary REE literature. Merely two approaches make explicit use of Bloom’s taxonomy to guide their instruction [19, 124] and only 10.5% of approaches (i.e., [4, 7, 11, 19, 40, 41, 53, 92, 104, 112, 127, 145, 155, 158, 160, 161, 176]) consider systematic pedagogy. Yet, with the exception of closely related studies such as [11, 40, 41], there seems to be no common pedagogical framework nor is there a common basis of systematically gathered evidence as to the effectiveness of teaching approaches given learning outcomes. In fact, to the best of our knowledge, the manuscript at hand is the first and thus far only systematic investigation into REE literature and students’ learning outcomes. We therefore declare a call to action for the REE community (and perhaps the software engineering education community at large) to produce a common, evidence-based pedagogical framework. We hope that the work at hand lays a suitable foundation for such an effort.

## Discussion, conclusions, and future work

In this paper, we presented the results of a systematic literature review into learning outcomes portrayed in Requirements Engineering Education (REE) literature. We have selected 152 primary studies from 1988 to 2020, to provide three contributions: (goal 1) We provide a systematic map of the current state of REE research. (Goal 2) We review the current practices and educational approaches to achieve learning outcomes. (Goal 3) We show how REE has changed in the last decade and which topics remain unexplored in the literature.

Our main findings include the recent trend towards authentic and industry-realistic learning experiences to improve students’ knowledge, predominantly on topics such as requirements elicitation and modeling, but also with regards to students’ soft skills, collaboration, teamwork, and industry-readiness. To accomplish this, current trends involve real or realistic stakeholders and role playing in low-stakes collaborative project-based instruction scenarios. Theory-based instruction plays a subordinate role in REE, suggesting that knowing about theory is less emphasized than effectively applying theory in industry-realistic settings, ideally spanning all parts of the RE process.

Our findings further suggest that areas where REE approaches are currently lacking include instruction of safety and security requirements engineering, as well as supply chain risk management. Moreover, REE presently suffers from a lack of a common pedagogical basis and systematically gathered evidence. While a plethora of successful teaching methods have been proposed (e.g., game-based learning, new frameworks, and educational tools), for the most part, these contributions are in isolation and not part of a systematic attempt to propose methods that are tailored to student outcomes.

We contrast and complement findings from a previous mapping study by Ouhbi et al. [135]. While Ouhbi et al.’s work focuses on REE approaches and their consideration of standardized curricula, we place emphasis on synthesizing learning outcomes and educational approaches reported in the literature. We also highlight developments in the field since Ouhbi et al.’s study concluded in 2012. In part, we were able to replicate Ouhbi et al.’s results, differ in some findings, and provide additional findings not previously reported.

To our knowledge, a replication of a systematic literature review or mapping study has thus far not yet been completed in the discipline of software engineering. While it was not our aim to replicate Ouhbi et al.’s work, we believe that the work at hand sufficiently highlights areas of overlap. This produces a secondary outcome of our work, i.e., that differences between our findings can be explained by differences in search methodology and as well as rigor in inclusion and exclusion criteria.

In this paper, we lay a foundation for the REE community to produce a rich evidence-based understanding of effective pedagogical approaches. Given the vastness of our data set, we envision future work focusing on qualitative analysis of previous studies to uncover new insights. For example, we found that interviews for elicitation is well studied in the literature. Future work could look at which other elicitation techniques are taught (e.g., questionnaires, analyzing competitors). Similarly, other studies could investigate how requirements quality metrics (e.g., correctness, consistency) are instructed.

In addition to studying the level of learners (see Sect. 4.9), future work could study these educational approaches with respect to which approaches are taught as part of introductory, intermediate, or advanced courses in RE and SE, at both the bachelors and master levels. This would give greater insight into the depth of RE curriculum, and would be complementary to initial efforts  [64, 67]. Supporting this line of inquiry, we also intend to survey educators to identify best practices and examine whether there are any instructional approaches that could be of relevance for RE education but have not been published.

As already introduced in Sect. 5, we found 33 papers (i.e., 21.7% of selected studies) with validated approaches, which was insufficient for our intended analysis. Given the importance of evidence as to the effectiveness of pedagogy, we seek to complete an in-depth qualitative analysis of these papers as part of future work in order to provide insights to what works and what does not work. By looking more deeply at RE activities, we can assist new educators in understanding what is recommended.

In addition, as already discussed in the paper (see Sects. 5.1, 5.2, and 6.1.4), we found the further research is required to explicitly investigate the relationship between industry-readiness and requirements proficiency among students. Finally, as proposed in Sect. 6.2.1, we need a systematic educational approach to instruct students on the development and importance of security requirements.

## Footnotes

1 Available at https://2021.refsq.org/details/refsq-2021-papers/3/The-Challenge-s-of-Teaching-Requirements-Engineering.

2 The data set is available here: 10.35482/csc.003.2021
